# Parental Provisioning in an Urban Apex Predator

**DOI:** 10.1002/ece3.72593

**Published:** 2025-12-04

**Authors:** Edward J. A. Drewitt, Brandon Mak, Innes C. Cuthill, Robert J. Thomas

**Affiliations:** ^1^ School of Biological Sciences University of Bristol Bristol UK; ^2^ Terrestrial Ecology Research Group, Department of Life Science Systems Technical University of Munich Freising Germany; ^3^ School of Biological Sciences Cardiff University Cardiff UK

**Keywords:** breeding ecology, citizen science, parental care, prey choice, urban wildlife

## Abstract

We combine high‐definition webcams and citizen science to explore parental care in urban peregrine falcons *Falco peregrinus* (hereafter ‘peregrines’) from 30 locations across England between 2019 and 2023. By analysing detailed webcam footage, we quantified changes in prey type and delivery rates as nestlings developed. Common starlings *Sturnus vulgaris* and pigeons *Columba livia* , both feral and domestic (loft), dominated the prey brought to the nest, with a progressive increase in the proportion of the larger species as nestlings aged. There was an initial increase in the rate of prey delivery from hatching until nestlings reached 9–12 days old, modulated to a degree by brood size, but thereafter delivery rates dropped progressively until fledging. Somewhat unexpectedly, there was no evidence for the total mass and energy supplied over the nestling period varying with brood size. This study provides valuable insights into the breeding ecology of urban‐dwelling peregrines and highlights the value of citizen science and technology in wildlife research.

## Graphical abstract

We combine high‐definition webcams and citizen science to explore parental care in urban peregrine falcons from 30 locations across England between 2020 and 2023. By analysing detailed webcam footage, we quantified changes in prey type and delivery rates as chicks developed.

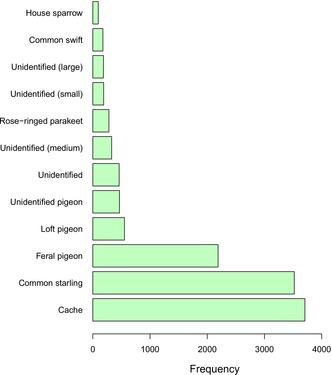

## 1 Introduction

Studying the breeding ecology of bird species can reveal important details on the phenology of their breeding cycles, parental provisioning and subsequent breeding success (Ydenberg 1994; Mock 2022). These in turn can both contribute fundamental knowledge to life‐history theory and help identify whether population change is due to changes in breeding productivity (number of nestlings fledged per adult) or not (Ricklefs and Bloom 1977; Ferguson‐Lees et al. 2011). Nest record data may also be used as part of a wider integrated population monitoring scheme, such as that used by the British Trust for Ornithology (BTO), to study bird population trends and help inform conservation and land‐management strategies (Baillie 1990; Ferguson‐Lees et al. 2011; Walker et al. 2023).

The breeding ecology of raptors (birds of prey) is of particular interest because many are positioned high in the food chain, often as superpredators or apex predators (Wallach et al. 2015). This makes their breeding productivity especially sensitive and vulnerable to changes occurring at lower trophic levels (Lourenço et al. 2011; Wallach et al. 2015). Although knowing how raptors provision their young is an important part of their natural history (Paviour 2013), their secretive behaviour, remoteness of their breeding locations and/or the rarity of some raptors means that this part of their life cycle is often the least understood. However, it can be vital for informing conservation measures that might be required to help raptor species that are declining or endangered, or whose populations are stable now but could decline in the future. Raptors living in urban locations are potentially easier to detect and monitor than those in rural locations, although research on urban wildlife remains relatively scarce but is increasing (Magle et al. 2012; Kettel et al. 2018; Leveau et al. 2022). So far, the provisioning of food for young has been studied in a range of urban‐breeding raptors (Sodhi and Oliphant 1993; Piattella et al. 1999; Cava et al. 2012; Kumar et al. 2014; Hindmarch and Elliott 2015; Riddell 2017; Solonen et al. 2019; Dykstra et al. 2003; Estes and Mannan 2003; Rutz 2004; Fisher 2020; Merling de Chapa et al. 2019; White et al. 2020, 2022; Thornton et al. 2025).

Although peregrines are well documented (Buechley et al. 2019), data on provisioning for young in urban breeding populations remain relatively scarce, and the study of their diet remains limited to small samples or use of indirect methods/sources (Rejt 2001, 2004; Drewitt and Dixon 2008; White et al. 2013; Dixon and Drewitt 2018; Kettel et al. 2016; Pettersen 2022; Sale and Watson 2024). The peregrine is therefore one such species whose study benefits from detailed nest record data and, being a charismatic apex predator, can help galvanise public involvement in, and support of, conservation (Drewitt 2014; Lorimer 2007; Searle et al. 2023). In a similar manner, urban‐breeding red‐tailed hawks *Buteo jamaicensis* in Reno‐Sparks, Nevada, USA, are viewed positively by 70% of local residents (White et al. 2018). In the United Kingdom, although peregrine numbers have increased across its range since the 1980s, its population appears to be decreasing in northern parts of England and Scotland (Wilson et al. 2018). This decline is thought to be largely due to illegal persecution, decline in prey species and degradation of associated habitats in rural areas (Wilson et al. 2018; Mak 2024). Despite this, peregrines are widespread across urban locations, particularly in the west, south and central areas of England, although their ecology has been much less extensively researched than that of their rural counterparts (Drewitt 2014; Mak et al. 2021; Drewitt et al. 2022; Adams et al. 2023). Although many animal species struggle to live in urban areas, peregrines appear to thrive. Urban‐breeding pairs produce a mean of one more nestling per breeding attempt than rural pairs and, although the diversity of bird species commonly eaten is lower in towns and cities, the overall density and biomass of bird prey are higher (Kettel et al. 2018). There is also evidence that anthropogenic influences, such as food subsidies on prey species such as feral pigeons, also indirectly benefit urban predators such as peregrines (Mak et al. 2023), potentially leading to their higher breeding success.

The growing popularity of web cameras at urban peregrine nests offers the possibility of far more detailed data on the provisioning of young. The term ‘technonatural history’ has been proposed to describe the role of new technologies in determining how people study and interact with a species (Searle et al. 2023). Searle et al. (2023) used the concept of the ‘digital peregrine’ as an example of how web cameras (at peregrine nests) have proliferated across the United Kingdom and how watching peregrines online connects more people with wildlife and provides a place where meaningful human–animal relationships can develop.

Social media platforms can also be used for ‘video mining’, systematically collecting videos that feature a particular species, such as the peregrine, for investigating their behaviours at the nest (Marziliano et al. 2023). In particular, during the COVID‐19 pandemic, opportunities arose for citizen science to play an important role in collecting data from streaming peregrine nest web cameras. Using volunteers in the wider community is becoming an efficient and positive means of engaging people with wildlife and collecting meaningful data, although it is important to ensure there is adequate training and/or quality control of data collection (Kosmala et al. 2016; Searle et al. 2023; Mak et al. 2023).

In our study, we use data collected from webcam nests by volunteers to analyse aspects of parental provisioning to test the following hypotheses:

Hypothesis 1 *Based on our previous study, we expected that the diet fed to the nestlings would be dominated by only two species of bird, common starlings (hereafter ‘starlings’) and feral or loft pigeons* (Mak et al. 2023); *in the Greater London area, rose‐ringed parakeets Psittacula krameri would also be expected to be important in the diet, this introduced species now being well established* (Heald et al. 2020; Mak et al. 2023).

Hypothesis 2 *Larger broods should receive a greater quantity and size of prey*.

Hypothesis 3 *Prey delivery should increase as nestlings age, with larger birds, such as pigeons, more likely to be delivered as the female, which is larger than the male, transitions from brooding the young chicks to hunting and prey delivery* (Olsen and Tucker 2003).

## 2 Methods and Materials

### 2.1 Study Locations

Between 2019 and 2023, we observed 30 different urban peregrine nests during the breeding season using live or recorded screen data from web cameras (Table A1 in Appendix 1). Although many of these nest locations are well known and publicised, the latitude and longitude for each location are represented at the lower resolution of two decimal places for reasons of secrecy because peregrines are still persecuted in some areas.

Each nest web camera had been set up independently by individuals or local groups, affiliated or non‐affiliated with the locations. As such, the sampling of nests was non‐random and the degree to which our data are representative of the behaviour of UK urban peregrines is unknown. There may be biases due to site accessibility and by the fact that local ornithologists may have chosen to site their webcams where pairs have a record of breeding successfully. However, we can say that our sample is geographically broad and the sampling at each site was intensive (24/7). We also acknowledge that any conclusions are based on an overall study period of 5 years (2019–2023), with most data coming from the middle 3 years of that period. We studied one location in 2019, followed by 16 in 2020, 21 in 2021, 24 in 2022 and 4 in 2023 (Table A1 in Appendix 1). This reflected the research team's capacity, with more volunteers recruited in 2021 and 2022. In 2023, data were obtained where they were already being collected by local individuals who were monitoring the web cameras independently. For this study, locations deemed urban were defined as those in towns and cities. One exception was the Cantley Sugar Factory which is a large rural industrial site located adjacent to a village (Cantley, some 15 km from Norwich, Norfolk).

Volunteers collected data from 17 urban locations that were streamed live on YouTube and where the previous 12 h could be reviewed directly using its playback function. The team found this the most accessible and easiest method to collect data. Volunteers recorded each feeding event as a screen recording onto their laptop or desktop computer using screen‐capture software already on the computer or free, open‐source software that could be downloaded. The most favoured were OBS Studio (obsproject.com) and QuickTime Player (Apple Inc., Cupertino, CA, USA). One location, Salisbury, was streamed through a host website and did not have a playback function. Therefore, the live streaming was screen recorded continuously and later reviewed. Further screen recordings were then taken to archive any prey deliveries. This method was less reliable as the live streaming sometimes failed or timed out and the webpage needed to be refreshed; meanwhile occasional feeds were missed. However, the amount of data collected was still comparable to other locations where all feeds were recorded.

For a further 12 locations, we collected data directly from network video recorders (NVR). These were places where live streaming across the internet was not possible and/or where there were historical data available from 2019 onwards that had not previously been used.

EJAD collected specific data referring to the size of broods and mass of nestlings—dependent on whether they were ringed (banded)—from the local observers, nest recorders or licenced ringers involved. All peregrine nestlings were ringed between 19 and 25 days old, within the recommended ringing period (Hardey et al. 2005). The sex of nestlings was determined during the ringing activity using a combination of biometrics, including mass, tarsus length and width, hind claw length and middle toe length (Hardey et al. 2005). Those nestlings with biometrics intermediate between the ranges for male and female peregrines remained unsexed. We defined brood size as the number of nestlings within a brood that made it to fledging. At three locations, one nestling died before reaching 3 weeks old; we excluded these nestlings from the total nestling numbers for those years and locations. However, analyses that include or exclude these nestlings—by changing the brood sizes they were part of—still gave the same overall results.

### 2.2 Identifying Prey

We were able to collect data from the web cameras over 24 h as the cameras included infrared illumination which allowed for nighttime peregrine activity to be observed. During the period of data collection, volunteers recorded and archived every prey item fed to peregrine nestlings at each location—regarded as a feeding event—as a screen recording (video clip) and included a timestamp on screen; they were stored in an online folder. Volunteers included the timing and description of the feeding event on a shared spreadsheet. The prey that featured in each screen recording was then reviewed and identity confirmed by EJAD who has extensive experience in identifying peregrine prey items (e.g., Drewitt 2020).

To correspond with each feeding event in the folder and spreadsheet, volunteers gave each screen recording a unique filename that reflected the location, date and sequentially numbered feeding event for that day. For example, CCR_010522_prey4 refers to Chichester on the 1 May 2022 and the fourth feeding event of the day. Although we began data collection when the eggs were laid, the analyses focused on the period between when the eggs hatched and when the nestlings fledged (in June/July) or were no longer in camera view. This was either because the nestlings had moved out of the camera's field of view or, in the case of one camera, it was covered in excrement.

We recruited volunteers in February, March and early April, at the beginning of each peregrine breeding season (March/April); they included biology students from the University of the West of England (UWE), the University of Bristol and Cardiff University and geography students from King's College London. Volunteers already monitoring specific locations also supplied data. EJAD and BM gave training to each cohort of volunteers by meeting online and providing handbooks on how to collect and archive the data.

When possible, EJAD identified prey to the lowest taxonomic level. In 18 cases (0.14%), it could not be determined whether a peregrine had brought in a prey item or not; these were left as missing values. In a further 324 cases (2.5%), a timestamp was not recorded for a feeding event but, from the position in the sequence of screen recordings, an approximate time could be interpolated. If the prey were unidentifiable, EJAD classified them into a size category (small, medium or large). When appropriate, they were labelled as cached prey; this enabled us to establish the overall proportion of cached prey fed to the nestlings to be established and provide an indication of its importance in the nestling diet. Cached prey refers to prey that has been hidden or put to one side and used to feed nestlings later. This was determined by EJAD distinguishing between new/fresh prey that had been fed to nestlings and prey that had been previously partially eaten by the nestlings before being taken away or hidden.

Although feral pigeons and loft pigeons belong to the same domesticated species of rock dove *Columba livia* , those lacking any indication of belonging to someone were labelled as feral pigeon. Loft pigeons, defined as those owned by someone, such as those used in racing competitions and white doves used in cultural ceremonies, were identified by their coloured leg rings, wing stamps and/or dyed feathers.

Although EJAD found that most prey items were identifiable to a species, some were not, usually because they had already been plucked or partially eaten before being brought to the nest. Fresh prey—if not identifiable to a species—was assigned by EJAD to a size category. Prey the size of feral pigeons or western jackdaws *Coloeus monedula* (hereafter ‘jackdaws’) were assigned to ‘unidentified (large)’; birds the size of common blackbirds *Turdus merula* (hereafter ‘blackbirds’) and starlings were assigned to ‘unidentified (medium)’; small sparrow‐ and finch‐size birds were assigned to ‘unidentified (small)’. Some prey, which had obviously been fed upon by the adults prior to being delivered, remained as ‘unidentified’. Plucked or semi‐plucked prey, especially if still uneaten, could often still be recognised as coming from a particular taxon and were, for example, labelled as ‘unidentified pigeon’, ‘unidentified corvid’ or ‘unidentified wader’. Labelling prey in this way—even if unidentifiable to a particular species—meant that an estimated mass could still be assigned to them, which enabled their use in the wider analyses of the data. Including unidentified prey in the analysis also allowed for the frequency of prey delivery to still be ascertained (Robinson et al. 2015).

The energetic value of prey was determined as 8.4 kJ/g (Lindberg 1983; Bird and Ho 1976), assuming that 100% of each prey item was eaten. Because we did not have biometric data on individual prey, the total energy per prey item was adjusted according to the average mass of that particular species or prey type. The energy content of prey eaten is the mass of the species or prey type multiplied by 8.4 so analyses for mass and energy content align with each other. We realise that while smaller prey, such as small passerines, may all be eaten in their entirety, larger prey, such as pigeons, may have wings and bones left uneaten; we also assumed the prey was 100% digestible and therefore acknowledge that our analyses may consistently overestimate the energy value of prey to the peregrines (Lindberg 1983). We adjusted the mass for a peregrine nestling and a pigeon squab to reflect their smaller size compared to an adult peregrine or pigeon (Darwati et al. 2010; Sale and Watson 2024).

### 2.3 Statistical Methods

We carried out all analyses using R4.4.0 (R Core Team 2024) with additional packages as specified below. Plots followed a colour‐blind friendly palette (Okabe and Ito 2008). We processed dates using the ‘lubridate’ package (Grolemund and Wickham 2011). We produced most graphs using the package ‘ggplot2’ (Wickham 2016), although some simple graphs were plotted using base R functions. We used the package ‘jpeg’ to read in images for embellishing some graphs (Urbanek 2021).

As there were no strong a priori predictions for most of the analyses (especially with regard to interactions), our analysis strategy was to fit a series of models of increasing complexity (from intercept‐only to highest‐order interaction) and then compare them using Akaike's Information Criterion (AIC; Burnham and Anderson 2002). AIC quantifies the balance between good fit, as measured by the likelihood, and complexity, in terms of the number of model parameters. In a comparison of models fitted to the same data, the model with the lowest AIC is the preferred model (best balance of good fit but low complexity), although, by convention, other models within 2 units of the ‘best’ model are considered equally plausible (Burnham and Anderson 2002). In all models, we included both ‘Nest ID’ (the breeding attempt in one place in 1 year) and ‘Location’ as random effects, to control for the fact that not only are data from the same nest non‐independent but nests in different years at the same location may not be statistically independent. This may be true even if the parents are not the same individuals in successive years, because breeding performance in different years may be correlated when the site (affecting, e.g., feeding opportunities) is the same. We also initially considered year as a random effect, but this explained no variation beyond that accounted for by Nest ID, leading to model convergence failures, so it is omitted from the analyses presented here. Where null hypothesis testing is of interest, we have provided significance tests, based on comparison of the deviance of a model with and without the term in question.

**FIGURE 1:**
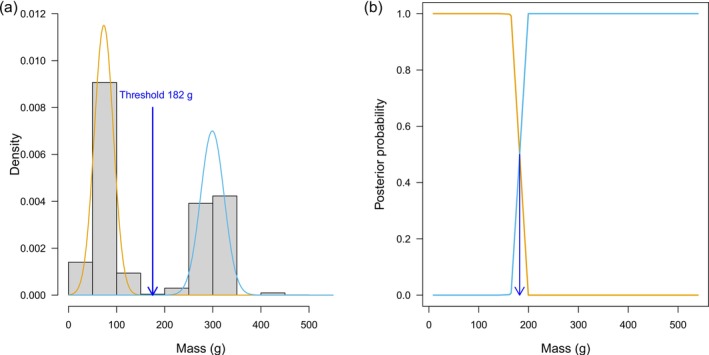
Gaussian mixture modelling of prey mass. (a) Histogram of prey masses (*n* = 8602 identified to species level and thus average mass available) with the two best‐fitting Gaussian distributions (orange and pale blue) and the threshold for classifying prey as ‘small’ or ‘large’ indicated by a blue arrow. The estimated means and standard deviations of the two distributions are 74 g (SD 20) and 299 g (SD 24). (b) The posterior probability from Gaussian mixture modelling of the two prey classes, ‘small’ and ‘large’ (orange and pale blue, respectively), with the threshold for the switch indicated by a blue arrow.

Prey mass (and correspondingly energy content) was bimodal, suggesting that there were basically two sizes of prey (‘starlings + smaller’ and ‘pigeon + other large’; see Figure 1). An objective criterion for the ‘small’ versus ‘large’ split was found by Gaussian mixture modelling: iteratively finding the best‐fitting Gaussian (normal) distributions, using the ‘normalmixEM’ function from the ‘mixtools’ package (Benaglia et al. 2009). The threshold for defining a prey item as ‘small’ or ‘large’ was the intersection point of the posterior probability distributions of the two fitted Gaussians, 182 g (Figure 1b). Thus, for analysis, we created a categorical variable for ‘prey size/energy’ with two levels (small and large) using the cut function in R to partition mass at the value 182 g. We analysed the potential effects on prey size of nestling age, brood size and their interaction with Hierarchical Generalised Additive Models (HGAMs), using the function ‘gam’ with a binomial error in the ‘mgcv’ package (Wood 2017; Pedersen et al. 2019). We similarly analysed changes in the proportion of feeding events that included cached prey with HGAMs and a binomial error. We used Generalised Additive Models in preference to Generalised Linear Mixed Models to allow for more complex non‐linearity in the relationship between prey size and date than could be achieved with a simple polynomial. All models included nest ID and location, modelled as random effect smoothers of the form ‘s(nest, bs = ‘re’) + s(location, bs = ‘re’)’ (Wood 2017; Pedersen et al. 2019). Generalised Additive Mixed Models, using the function ‘gamm’, produced very similar estimates, but we present the HGAM results on account of the greater numerical stability of the algorithms used for model fitting (Pedersen et al. 2019).

The number of daily feeding events was seen, a posteriori, to increase and then decrease again as the nestlings aged. Differences in the pattern of change among brood sizes were also examined with HGAMs, again using the function ‘gam’, this time with a Poisson error and log link (Wood 2017; Pedersen et al. 2019). Again, all models included nest ID and location, modelled as random effect smoothers.

We analysed the total energy brought to the nestlings in each nest over the nestling‐rearing period (i.e., summed from first to last date of food delivery) with respect to brood size with a linear mixed model (LMM), using the function ‘lmer’ from the ‘lme4’ package, including location as a random effect (Bates et al. 2015). As prey were not identified for all feeding events (either because the item could not be observed clearly or because it was a previously cached item and dismembered), we estimated total energy delivery in three ways: (1) using only feeding events in which prey were definitively identified or the mass classifiable to small, medium or large (see earlier); (2) assuming that prey for feeding events where the item was not observed, or it was a cached item, had the same average energy content as identified prey; (3) as in (2) but omitting cached prey. Because re‐delivered cached items consisted of remnants of prey left over from a previous feeding, it seems likely that cached prey had lower energy values than the average freshly caught prey at a given nest.

The total number of days for which data was available for a given nest was expected to affect total energy delivered over the nestling‐rearing period, and the data for some nests stopped short of the expected time of fledging. This could have been due to parents switching their feeding location towards fledging, such that the camera was not triggered, or genuine early fledging. To account for this, we included nestling‐rearing period as a covariate (i.e., a mixed‐model ANCOVA), testing for the parallel‐slopes assumption for the effects of nestling‐rearing period with respect to brood size by means of the brood size by nestling‐rearing period interaction.

Because nestlings were ringed near fledging, we were able to compare nestling masses among brood sizes. To do this, LMMs were fitted with the predictors sex (male, female and unsexed) and brood size, and random effects ‘Nest ID’ and ‘Location’. The sample sizes for nestling mass were lower than for the other analyses (75 nestlings in 29 nests across 20 locations).

## 3 Results

### 3.1 Prey Types

**FIGURE 2:**
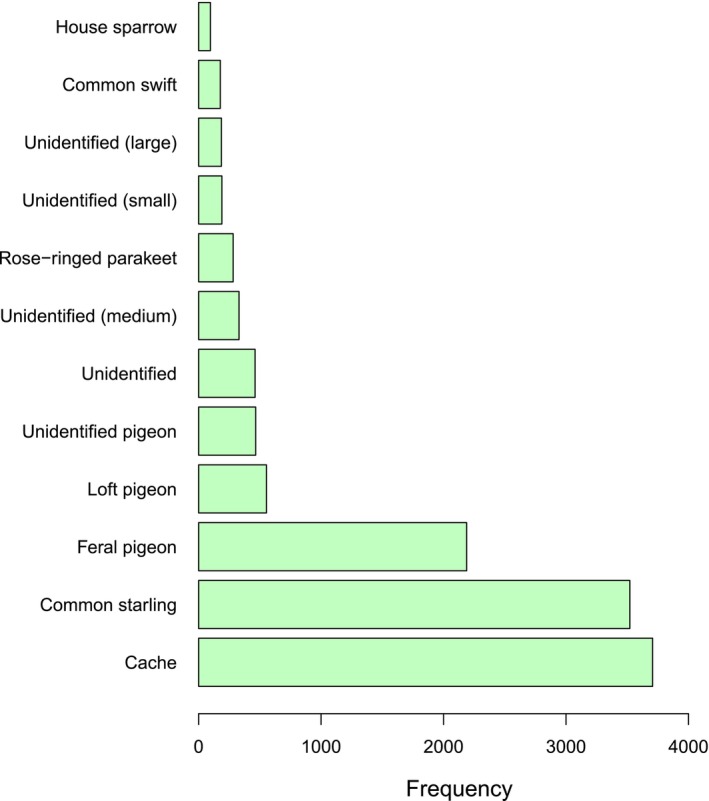
The frequency of the top 12 prey items that account for 95% of the prey observations across all locations and years (total number of observations = 12,771).

**FIGURE 3:**
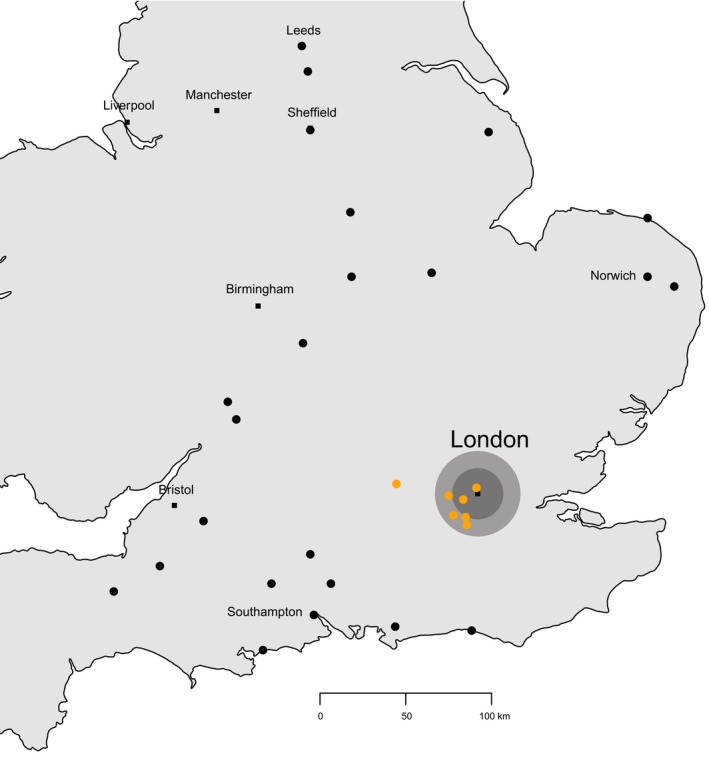
Distribution of peregrine nests in which rose‐ringed parakeets were fed to nestlings (orange dots) or not (black dots). A selection of cities (black squares) is indicated for orientation. The nests with parakeets in the diet were in south‐west London (Charing Cross, Ealing, Kingston, London Metropolitan University, Merton Civic Centre and Sutton) or, a short distance to the west of London (Marlow).

Across the four breeding seasons, 12,771 feeding events were observed from the web cameras during the nestling stage, 0–50 days. Of these, 3707 (29%) feeding events comprised cached prey and 9064 (71%) involved new, fresh prey items. In the latter, we identified 70 species of bird (Tables A2 and A3 in Appendix 1) alongside one unidentified bat and a field vole *Microtus agrestis* . Starlings (28%) and pigeons (25%, comprising 17% feral, 4% loft, 4% unidentified pigeon (either *Columba* spp. or *Streptopelia* spp.)) were by far the most common species in the diet (Figure 2; Tables A2 and A3 in Appendix 1); the next most frequent prey species were rose‐ringed parakeets (3%)—fed to nestlings at sites in south‐west London and surrounding regions (Figure 3)—and common swifts *Apus apus* (hereafter ‘swifts’) (1%). Because of the prevalence of starlings and pigeons, the prey effectively fell into two main size categories and thus energetic content (Figure 1): small‐to‐medium (starling‐ or parakeet‐size and below) and large (pigeon‐size and above). Excluding cached prey, nearly 66% of the energy supplied to nestlings, therefore came from pigeons (45% feral, 11% loft and 10% unidentified pigeons) and 18% from starlings. The largest prey observed were two Eurasian oystercatchers *Haematopus ostralegus* (ca. 580 g, both in the Taunton nest) and the smallest were two common chiffchaff or willow warbler *Phylloscopus collybita* or *P. trochilus* (ca. 9 g, one in Leicester, one in Marlow).

**FIGURE 4:**
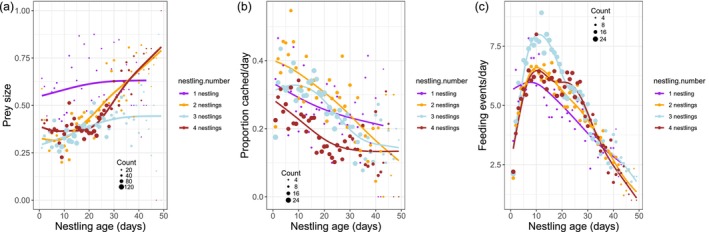
Change as a function of nestling age in (a) the proportion of large (vs. small) prey; (b) the proportion of feeding events that involved cached items as opposed to freshly caught prey; (c) the number of feeding events per day. Lines were generated from the best‐fitting HGAM models for each brood size (see Table 2).

Modelling prey size as a binary variable (large vs. small) to determine how diet changed as a function of nestling age, the best‐supported model included the interaction between brood size and nestling age (Table 1). We investigated what underlay this interaction by analysing each brood size separately. Nests with single nestlings received a high (60%–65%) and relatively consistent proportion of larger prey as nestlings aged (the effect of nestling age was not statistically different from zero; Table 1). Conversely, parents of larger broods delivered relatively more small prey to young nestlings and then the proportion of large prey increased as nestlings aged (Table 2, Figure 4a).

**TABLE 1:** Comparison of models for the change in prey size (small vs. large), the proportion of cached items and feeding events per day, as a function of nestling age and brood size.

Predictors	Prey size				Proportion cached items				Feeding events/day			
	LogLik	df	AIC	d.AIC	LogLik	df	AIC	d.AIC	LogLik	df	AIC	d.AIC
Age × brood.size	−4993.9	81.1	10,150.0	0.0	−2845.5	72.5	5835.9	0.0	−5039.9	87.7	10,255.2	0.0
Brood.size	−5013.2	68.3	10,162.9	13.0	−2856.4	66.1	5844.9	9.0	−5060.8	70.9	10,263.4	8.1
Age + brood.size	−5012.9	68.7	10,163.2	13.2	−2856.1	65.6	5843.3	7.4	−5060.9	71.0	10,263.8	8.6
1	−5138.4	61.8	10,400.4	250.5	−2950.6	63.1	6027.4	191.5	−5672.8	63.0	11,471.5	1216.3
Age	−5138.1	62.2	10,400.6	250.7	−2950.3	62.6	6025.8	189.9	−5673.0	63.1	11,472.0	1216.8

**TABLE 2:** Separate models for each brood size for the effect of nestling age on the proportion of large (vs. small) prey, proportion of cached prey and the number of feeding events per day.

Brood size	Prey type			Proportion cached items			Feeding events/day		
	edf	Chi.sq	*p*	edf	Chi.sq	*p*	edf	Chi.sq	*p*
1 nestling	1.55	4.35	0.077	1.75	6.67	0.049	3.38	70.96	< 0.001
2 nestlings	4.34	113.45	< 0.001	1	53.21	< 0.001	6.36	242.47	< 0.001
3 nestlings	3.18	55	< 0.001	1	57.07	< 0.001	7.26	527.48	< 0.001
4 nestlings	3.57	89.49	< 0.001	3.32	83.13	< 0.001	6.46	270.91	< 0.001

The proportion of feeding events in which parents delivered previously cached items was very low when nestlings were 1 day old, presumably because few prey items had been cached prior to this. The day 1 values were distinct outliers compared to the subsequent pattern of change as nestlings grew, so the day 1 values were omitted from the models presented here. By day 2, there was an abrupt increase in feeding events involving cached prey, followed by a steady decline with nestling age, although the pattern of variation apparently differed among brood sizes (Tables 1 and 2; Figure 4b). For broods of one, two and four nestlings, there was a fairly steady decline in the proportion of cached items with nestling age, but in broods of three nestlings, there was an initial increase, peaking when the nestlings were around 12 days old. Note also a similar peak in feeding event rates at this age for broods of three (Figure 4c; see later).

### 3.2 Feeding Events

The mean number of feeding events per day increased up to ca. 12 days and then declined as the nestlings developed (Figure 4c). Although the pattern of change was broadly similar across brood sizes, a model including the interaction among brood size and nestling age was better supported than a model with main effects only (Table 1). The feeding event rate for single nestlings tended to peak slightly earlier (age 9 days) and drop faster than for larger broods, with the highest feeding event rate for broods of three, observed at around 12 days of age (Figure 4c). A higher feeding event rate was maintained for longer for broods of four.

### 3.3 Total Energy Supplied to Each Nest Over the Nestling‐Rearing Period

**FIGURE 5:**
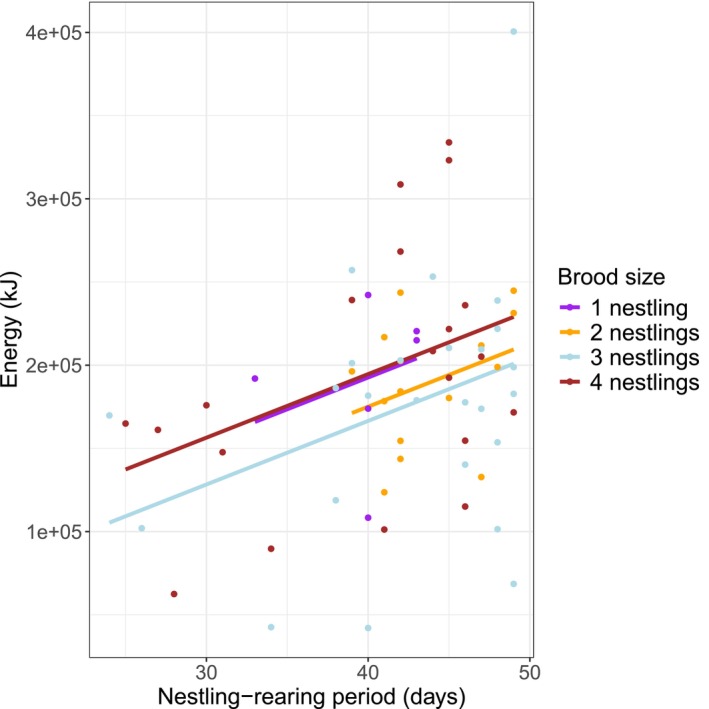
The relationship between the total energy of prey supplied over the nestling‐rearing period and the length of that nestling‐rearing period (the number of days for which prey were recorded being delivered to that nest), split by brood size. Brood size 1 *n* = 6, brood size 2 *n* = 15, brood size 3 *n* = 25, brood size 4 *n* = 20. The lines are the best‐fit linear regressions from LMM, assuming parallel slopes for the different brood sizes (justified by the non‐significant brood size by period interaction; see main text).

Analysing only feeding events where prey were identified, there was no detectable relationship between the total energy delivered to a nest and the brood size within it (LMM: *χ* ^2^ = 3.74, df = 3, *p* = 0.291). This remained the case even after controlling for the positive relationship between nestling‐rearing period and the total amount of energy delivered as prey (Figure 5; LMM: nestling‐rearing period *χ* ^2^ = 11.78, df = 1, *p* < 0.001; brood size *χ* ^2^ = 5.64, df = 3, *p* = 0.131; testing the parallel slopes assumption: *χ* ^2^ = 4.93, df = 3, *p* = 0.177). If we assume that all feeding events where prey were unidentified involved prey of the same average energy content as prey brought to that nest that were identified, we can adjust the estimated total energy brought to each nest using the ratio of total feeding events to feeding events where prey were identified. The conclusion that there was no detectable difference in energy brought to different brood sizes remained the same (nestling‐rearing period *χ* ^2^ = 1.66, df = 1, *p* = 0.197; brood size *χ* ^2^ = 0.15, df = 3, *p* = 0.986; testing the parallel slopes assumption: *χ* ^2^ = 2.16, df = 3, *p* = 0.539). Repeating this analysis but omitting feeding events involving cached prey produced similar results (nestling‐rearing period *χ* ^2^ = 12.75, df = 1, *p* < 0.001; brood size *χ* ^2^ = 4.32, df = 3, *p* = 0.229; testing the parallel slopes assumption: *χ* ^2^ = 2.58, df = 3, *p* = 0.460).

### 3.4 Nestling Mass, Sex and Brood Size

**FIGURE 6:**
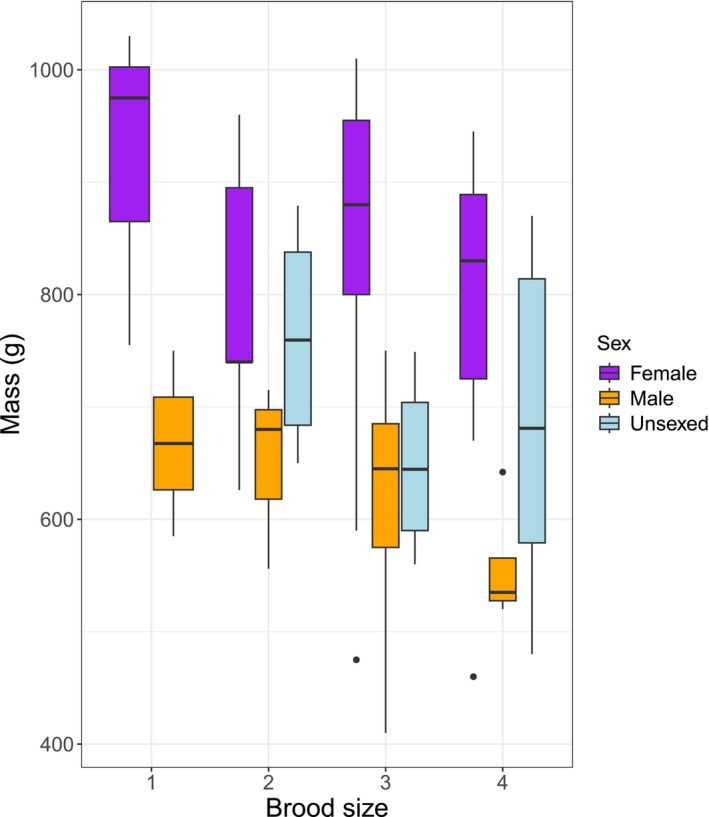
Nestling body mass differences with respect to brood size, while accounting for the large difference in mass between the sexes. Brood size 1: Female *n* = 3, male *n* = 2, unsexed *n* = 0; brood size 2: Female *n* = 5, male *n* = 3, unsexed *n* = 4; brood size 3: Female *n* = 17, male *n* = 21, unsexed *n* = 4; brood size 4: Female *n* = 7, male *n* = 4 and unsexed *n* = 5.

There was no sex ratio bias in nestlings with respect to brood size (48% male; Fisher's exact test, two‐tailed, *p* = 0.631; see Figure 6 legend for *n*). The mean mass for female nestlings was 828.1 g (*n* = 32); for male nestlings, it was 625.9 g (*n* = 30). Thirteen nestlings remained unsexed and, as one might expect, their masses were intermediate (mean 697.7 g) (Figure 6). In the GLMM analysis of nestling body mass, there was no interaction between sex and brood size (GLMM: *χ* ^2^ = 1.29, df = 5, *p* = 0.936), the average effect of brood size was not significant (main effect of brood size: *χ* ^2^ = 2.89, df = 3, *p* = 0.409) but, as expected, the main effect of sex was significant (*χ* ^2^ = 67.58, df = 2, *p* < 0.001). Treating brood size as a continuous variable did not change the conclusion (*χ* ^2^ = 1.03, df = 1, *p* = 0.311; slope = −19.4, 95% CI −60.7 to 18.6). Note that this analysis includes unsexed nestlings as a separate category of ‘sex’, but excluding such nestlings did not change the result (*χ* ^2^ = 2.02, df = 1, *p* = 0.155; slope = −30.7, 95% CI −74.1 to 12.5).

## 4 Discussion

This study of urban breeding peregrines provides a detailed analysis of the parental provision given to dependent young, with a focus on changes in their diet and energy consumption over time and among brood sizes. The use of high‐definition web cameras and ‘citizen science’ volunteers between 2019 and 2023 has provided the opportunity to study urban‐dwelling peregrines in ways that would have once been unfeasible.

### 4.1 Prey Species

Although the positively identified prey fed to nestlings included some 70 species, the diet of urban peregrines was dominated by starlings and pigeons, the former the commonest by number but the latter contributing 66% of the energy supplied to nestlings. In south‐west London and nearby regions, rose‐ringed parakeets were also an important (starling‐size) prey item. This dominance by a small number of species is also shared by other urban raptors such as Cooper's hawks *Astur cooperii* and Eurasian goshawks *Astur gentilis* (Rutz 2004; Cava et al. 2012). These results are also similar to those of some non‐urban diet studies where peregrines appear to specialise in a small number of species (Hunter et al. 1988; Olsen and Tucker 2003; Dixon et al. 2018).

Elsewhere peregrines are often regarded as a generalist predator (Ratcliffe 1993; Kettel et al. 2018), for example, in northern Spain breeding pairs hunted a wide range of diets of migratory birds (Zuberogoitia et al. 2013). Their specialist hunting in urban locations may reflect the fewer species in this environment compared to rural areas, although those species can be present in high numbers and densities (Kettel et al. 2018).

Nevertheless, the fact that 70 species of birds were recorded in total across the locations does suggest some opportunistic hunting. Indeed, at some urban locations, species other than pigeons, rose‐ringed parakeets, starlings, house sparrows and swifts do feature in the top three prey items (by frequency) (Mak et al. 2023). For example, the study by Mak et al. (2023) found that common tern *Sterna hirundo* , jackdaw, Eurasian chaffinch *Fringilla coelebs* and blackbird each separately feature in the top three prey species for different individual sites, respectively. This may be because they are locally abundant, easier to catch compared to other species (and therefore may be taken more frequently than would be expected by their population density), provide greater nutritional content compared to other species or be the preferred prey of individual peregrines (Dixon et al. 2018; Mills et al. 2019; Sale and Watson 2024).

Peregrines with single nestlings delivered a similar proportion of larger prey (i.e., pigeons) at all nestling ages; indeed, for younger nestlings, this represented a higher proportion than for parents with larger broods (Figure 4a). Conversely, birds with broods of 2–4 nestlings initially focused on delivering smaller prey items during the first 2–3 weeks after hatching. These differences among brood sizes could be due to a range of factors such as parental experience and health, availability of prey species, weather or competition with other peregrines (including their partners) or other raptor species (Newton 1979).

As the nestlings grew, the proportion of larger prey in the diet increased. The daily feeding event rates for single nestlings were somewhat lower than for larger broods, so there seems to be a different balance in the trade‐off between feeding event rate and prey size (McKinnon, Hawkshaw, et al. 2024). Parents of single nestlings give fewer feeds but deliver larger prey. Larger prey like pigeons can provide a substantial amount of energy compared to a medium‐sized bird such as a starling (Lindberg 1983), so one might expect these to be preferred. Catching fewer yet larger prey items for their nestlings may allow the parents to allocate more time to other activities such as protecting the nest and tending the nestlings (McKinnon, Hedlin, et al. 2024). Nevertheless, the hatching of peregrine nestlings generally coincides with the period when young starlings fledge (BTO 2024a, 2024b). Young starlings emerge from the nest ca. 21 days after hatching; being young and inexperienced, with their sudden appearance in the environment, and being more common, naïve and flocking than many other urban birds, they are probably easier for peregrines to catch compared to pigeons (Feare 1984). However, as we only have data on what was delivered to the nest, we cannot tell the extent to which these differences in diet (and among brood sizes) are driven by external factors, such as the availability of different prey species, and the differing demands of the brood.

Other studies of raptors also show how the diet changes as the nestlings mature. For example, lesser kestrels (*Falco naumanni*) primarily hunt saddle‐backed bush crickets (*Ephippiger ephippiger*) during their incubation phase and switch to hunting mostly migratory locusts (*Locusta migratoria*) and white‐faced bush‐crickets (*Decticus albifrons*) when they have nestlings; the diversity of prey decreases as their nestlings get older, whereas the biomass of their prey increases (Rodríguez et al. 2010). This is also reflected in crowned eagles (*Stephanoaetus coronatus*) at the other end of the size spectrum. In South Africa, adult crowned eagles feed their young mostly rock hyraxes (*Procavia capensis*) and hadeda ibises (*Bostrychia hagedash*). However, as they get older, and probably coinciding with when the female eagle begins to hunt, they start bringing back larger prey, in particular, vervet monkeys (*Chlorocebus pygerythrus*) (Van der Meer et al. 2018). In red‐tailed hawks, both bird and mammal prey increased in frequency as the breeding season progressed; those nesting in more urban locations had a greater diet breadth and fed their nestlings more birds, while those in more suburban areas were fed more mammals (White et al. 2022). Unlike, the peregrine, mammal specialists such as the common kestrel (*Falco tinnunculus*), nesting in the city centre of Vienna, Austria, face challenges hunting small mammals due to a lack of grassland habitat, leading to lower breeding success. The inner‐city kestrels have to hunt more bird prey and although they fly out of the city centre to find more suitable habitat to hunt small mammals, these comprise a smaller proportion of the diet compared to their suburban counterparts (Sumasgutner et al. 2014).

In our study, the importance of rose‐ringed parakeets in the diet of peregrine nestlings in south‐west London and nearby regions adds further to our knowledge of how the availability of introduced prey species affects how raptors provision their nestlings (Speziale and Lambertucci 2013). Naturalised bird species may be more accessible and available in larger numbers to raptors, particularly in urban locations, than naturally occurring species (Palma et al. 2006; Rodríguez‐Pastor et al. 2012; Butler et al. 2013; Feng and Himsworth 2014; Hancock and Martin 2015; Pârâu et al. 2016). In urban‐breeding Cooper's hawks in British Columbia, Canada, the introduced species house sparrow and starling made up half the diet (Cava et al. 2012). In Saskatoon in Canada, house sparrows made up 64.5% by frequency and 55.5% by mass of the diet of urban‐breeding merlins *Falco columbarius* (Sodhi and Oliphant 1993). For red‐tailed hawks nesting in Reno‐Sparks, Nevada, those living closer to urban centres provisioned their nestlings with a higher proportion of bird prey, particularly introduced feral pigeons and starlings, compared to those in suburban areas (White et al. 2022).

### 4.2 Feeding Event Rates

Feeding event rates to the nest initially increased as the nestlings grew and, although one might have expected feeding event rates to continue to increase with nestling age (Olsen and Tucker 2003), they instead peaked and declined from ca. day 12 onwards, with that decline coming slightly later for broods of four. We note that most of the early provisioning is by the male and during this period he is feeding both the nestlings and his mate. The drop in feeding event rate after the weeks 1–2 peak coincides with the period when female peregrines typically begin hunting also, so deliveries to the nest are then only for feeding the nestlings (Olsen and Tucker 2003; Sale and Watson 2024; McKinnon, Hawkshaw, et al. 2024). The female generally begins hunting once the nestlings can thermoregulate for themselves and do not need to be continually kept warm or sheltered by her (Ratcliffe 1993). Some evidence suggests that both sexes catch similar‐size prey and that even when the female stops brooding the young, the male still does most of the hunting (Zuberogoitia et al. 2013).

### 4.3 Parental Care and Caching

This study also highlights the importance of cached prey in the diet of urban peregrine nestlings, although how caching is influenced by the availability of prey and the differing demands of the brood is also unknown. The cached prey made up a significant part (on average nearly 30%) of the nestlings' diets, although the percentage dropped for older nestlings. There is surprisingly little in the literature to compare this figure to, as many studies report prey data from nests retrieved as physical prey items or pellets rather than detailed observations from web cameras (Fox 1979; Cameron and Olsen 1993; Drewitt 2014, 2020, 2024). However, Cameron and Olsen (1993) monitored a single nest across the nestling development stage and recorded prey being cached on 29 occasions (and retrieved 27 times), particularly when the nestlings were very young. There is no indication of what proportion this was of the total number of prey items fed to the nestlings; prey delivery is instead presented as a rate per hour.

### 4.4 Declining Prey Species

The prey data provides a snapshot of the urban peregrines' diet in the early 21st century. However, the significant decline in starling populations (54% since 1995) raises concerns about the long‐term implications for peregrine breeding success (Heywood et al. 2024). If starling numbers continue to dwindle, peregrines may face challenges in providing sufficient food for their young. This could lead to reduced nestling survival or a shift in prey preferences, potentially towards pigeons or other available bird species.

The availability of prey can significantly impact peregrine breeding success. For instance, a study in South Wales has linked high peregrine densities to higher racing (loft) pigeon populations (Dixon et al. 2003). Since this study, a decline in the population of racing pigeons, due to changes in racing routes, is thought to be the reason for a decrease in the number of breeding peregrine pairs in Central Wales due to reduced food availability and low breeding success (Dixon et al. 2010). Pairs here do not appear to have switched diet and seem to be specialist hunters, perhaps because there is not the prey availability—such as starlings and redwings *Turdus iliacus* —that they have in the winter. In contrast, breeding peregrines in northern Spain feed on a wide range of migrant birds (Zuberogoitia et al. 2013). In addition, research has demonstrated that increased pigeon availability can boost peregrine breeding productivity in Spain (López‐López et al. 2009). Although current evidence suggests that urban peregrines in the United Kingdom have sufficient prey, continued monitoring is essential to assess the potential impacts of ongoing environmental changes (Kettel et al. 2018).

### 4.5 Brood Size and Energy Delivered

Unexpectedly, we found no significant difference in total energy delivered over the breeding period among different brood sizes. Larger broods have more mouths to feed, so more food would be thought to be required to fulfil their energy requirements. However, similar results have been documented in other studies of peregrines (Olsen et al. 1998; Boulet et al. 2001) and Eurasian sparrowhawks *Accipiter nisus* , whose diets also comprise mostly birds (Newton 1986). One explanation is that the parents of larger broods may already be catching as much food as possible in the local environment, so are having to spread food more thinly between the nestlings. We found no significant differences in nestling mass to support this interpretation, but we can have no confidence in a null effect. Our sample size for nestling mass was small and the corresponding confidence intervals around the estimated slope of mass on brood size were very large (slope = −19.4, 95% CI −60.7 to 18.6).

### 4.6 Limitations

Despite the high‐resolution capabilities of modern web cameras, the process of streaming footage over the internet often results in significant quality degradation. This compression can hinder prey identification, leading to more generic labels like ‘unidentified pigeon’. Although analysing raw footage from network video recorders would provide a more accurate dataset, it is a less practical approach, requiring physical access to the recording equipment. This would limit the potential for widespread citizen science participation.

Correctly identifying prey species requires specialised knowledge and expertise. When this expertise is limited, prey items may be misidentified or categorised into general size classes, especially if partially consumed or obscured. Although this study benefited from EJAD's expert identification and quality control, a portion of the prey items could only be assigned to size groups due to damage or incomplete prey items. Nevertheless, this data remains valuable for understanding the overall diet and foraging behaviour of urban peregrines.

When studying the diet of adult urban peregrines, prey consumed away from the nest may go undetected leading to a bias in what is seen or found and therefore affecting what prey is recorded. Our study minimised these biases by focusing on nestling feeding behaviour only. Every feeding event was captured on camera, regardless of prey size or type. This comprehensive approach allowed us to obtain an accurate representation of the diet of peregrine nestlings.

## 5 Conclusions

By using streaming and offline web cameras, we have been able to acquire detailed quantitative samples of data to assess the breeding ecology of urban‐nesting peregrines. The ability to do this on a large scale has been made possible by using a citizen science model, managing volunteers who spent time observing and recording behaviours.

It reveals that their diet is largely composed of just three or four species of bird and aligns with a study by Kettel et al. (2019) where, despite urban locations having a lower diversity of bird species, there is still an abundance of food. We have found that food provisioning increases at similar rates across different brood sizes, and there are no significant differences among broods in overall energy provisioning. The results emphasise the importance of both feral pigeon and starling in the urban peregrine diet and highlight how the conservation of the starling, currently in decline in the United Kingdom, has the potential to affect the breeding success of predators such as the peregrine.

Urban‐breeding peregrines in the United Kingdom have one of the highest success rates of any raptors studied (Kettel et al. 2019). This study contributes towards our knowledge of urban raptor breeding behaviour and provides insights into why urban‐breeding peregrines are breeding successfully in our towns and cities and could be further compared to those of rural breeding peregrines, particularly those that are declining in parts of northern England and Scotland (Wilson et al. 2018). If conservation actions are needed in the future, this study contributes towards increasing our understanding of the peregrines' ecology (Buechley et al. 2019). It also contributes towards our continuing collective knowledge of urban raptors as encouraged by White et al. (2020): ‘As urban expansion continues… we stress that researchers monitor reproductive output across the urban predator guild to elucidate patterns in population dynamics and adaptation’.

## Author Contributions

**Edward J. A. Drewitt:** conceptualization (lead), data curation (equal), formal analysis (equal), writing – original draft (lead), writing – review and editing (equal). **Brandon Mak:** data curation (equal), methodology (equal), writing – review and editing (supporting). **Innes C. Cuthill:** formal analysis (equal), supervision (lead), writing – review and editing (equal). **Robert J. Thomas:** formal analysis (supporting), supervision (supporting), writing – review and editing (supporting).

## Disclosure

Statement of Inclusion: Our study was based in the United Kingdom and involved the engagement of both researchers and volunteers from across the country including the towns and cities in which the nest web cameras were based.

## Conflicts of Interest

The authors declare no conflicts of interest.

## Data Availability

Data are available at the University of Bristol data repository, data.bris, at https://doi.org/10.5523/bris.304bpwxi91gaj2amhjbjlvyroc.

## Appendix 1

**TABLE A1:** The locations of the urban peregrine nests studied alongside their latitude, longitude and the years they were monitored.

Location	Latitude	Longitude	Years recorded
Andover	51.21	−1.48	2020, 2021, 2022
Bath	51.38	−2.36	2020, 2021, 2022
Bournemouth	50.72	−1.87	2020
Brighton	50.82	−0.15	2020, 2022
Cantley	52.58	1.52	2021
Charing Cross	51.49	−0.22	2020, 2021, 2022, 2023
Cheltenham	51.9	−2.09	2021
Chichester	50.84	−0.78	2020, 2021, 2022
Cromer	52.93	1.3	2020, 2022
Ealing	51.51	−0.34	2022
Glastonbury	51.15	−2.72	2022
Kingston	51.41	−0.3	2020, 2021, 2022
Leamington Spa	52.29	−1.54	2020, 2021, 2022
Leeds	53.81	−1.55	2022
Leicester	52.63	−1.14	2022, 2023
London Metropolitan University	51.55	−0.11	2020, 2021, 2022
Louth	53.37	−0.01	2021, 2022
Marlow	51.57	−0.77	2021, 2022
Merton Civic Centre	51.4	−0.2	2020, 2021, 2022
Norwich	52.63	1.3	2019, 2021
Nottingham	52.96	−1.15	2020, 2021, 2022
Salisbury	51.06	−1.8	2020, 2021
Sheffield	53.38	−1.48	2020, 2021, 2022
Southampton	50.9	−1.45	2022
Stamford	52.65	−0.48	2022
Sutton	51.36	−0.19	2021, 2022
Taunton	51.02	−3.1	2020, 2021, 2022
Tewkesbury	51.99	−2.16	2021, 2022, 2023
Wakefield	53.68	−1.5	2020, 2021, 2022, 2023
Winchester	51.06	−1.31	2021

**TABLE A2:** Prey species or type and their frequency, percentage of total and cumulative percentage of total (*n* = 12,771).

Species/type		Frequency	% of total	Cumulative %
Cache	N/a	3707	29.03	29.03
Common starling	*Sturnus vulgaris*	3521	27.57	56.60
Feral pigeon	*Columba livia*	2189	17.14	73.74
Loft pigeon	*Columba livia*	554	4.34	78.08
Unidentified pigeon	*Columba* spp. or *Steptopelia* spp.	466	3.65	81.72
Unidentified	N/a	462	3.62	85.34
Unidentified (medium)	N/a	330	2.58	87.93
Rose‐ringed parakeet	*Psittacula krameri*	282	2.21	90.13
Unidentified (small)	N/a	190	1.49	91.62
Unidentified (large)	N/a	186	1.46	93.08
Common swift	*Apus apus*	177	1.39	94.46
House sparrow	*Passer domesticus*	96	0.75	95.22
Eurasian collared dove	*Streptopelia decaocto*	64	0.50	95.72
Western jackdaw	*Coloeus monedula*	56	0.44	96.16
Unidentified corvid	*Corvus* spp. or *Coloeus* sp.	56	0.44	96.59
Common blackbird	*Turdus merula*	50	0.39	96.99
Common wood pigeon	*Columba palumbus*	40	0.31	97.30
Great spotted woodpecker	*Dendrocopos major*	27	0.21	97.51
Common tern	*Sterna hirundo*	23	0.18	97.69
European goldfinch	*Carduelis carduelis*	21	0.16	97.85
European greenfinch	*Chloris chloris*	19	0.15	98.00
Eurasian chaffinch	*Fringilla coelebs*	15	0.12	98.12
Stock dove	*Columba oenas*	13	0.10	98.22
Common moorhen	*Gallinula chloropus*	12	0.09	98.32
White wagtail	*Motacilla alba*	11	0.09	98.40
Rook	*Corvus frugilegus*	11	0.09	98.49
Great tit	*Parus major*	10	0.08	98.57
Unidentified wader	*Calidris* spp. or *Arenaria* sp.	10	0.08	98.65
Red knot	*Calidris canutus*	9	0.07	98.72
Black‐headed gull	*Chroicocephalus ridibundus*	8	0.06	98.78
Dunlin	*Calidris alpina*	7	0.05	98.83
Western house martin	*Delichon urbicum*	7	0.05	98.89
Squab	*Columba* spp.	7	0.05	98.94
Eurasian jay	*Garrulus glandarius*	6	0.05	98.99
Sanderling	*Calidris alba*	6	0.05	99.04
Common redshank	*Tringa totanus*	5	0.04	99.08
Common ringed plover	*Charadrius hiaticula*	5	0.04	99.12
Common snipe	*Gallinago gallinago*	5	0.04	99.15
Unidentified thrush	*Turdus* spp.	5	0.04	99.19
Black‐tailed godwit	*Limosa limosa*	4	0.03	99.22
Eurasian blackcap	*Sylvia atricapilla*	4	0.03	99.26
Common cuckoo	*Cuculus canorus*	4	0.03	99.29
Common kingfisher	*Alcedo atthis*	4	0.03	99.32
Little grebe	*Tachybaptus ruficollis*	4	0.03	99.35
Eurasian magpie	*Pica pica*	4	0.03	99.38
Mistle thrush	*Turdus viscivorus*	4	0.03	99.41
Common quail	*Coturnix coturnix*	4	0.03	99.44
Eurasian skylark	*Alauda arvensis*	4	0.03	99.48
Song thrush	*Turdus philomelos*	4	0.03	99.51
Unidentified passerine	Passeriformes	4	0.03	99.54
Eurasian blue tit	*Cyanistes caeruleus*	3	0.02	99.56
Northern lapwing	*Vanellus vanellus*	3	0.02	99.58
Peregrine egg	*Falco peregrinus*	3	0.02	99.61
Peregrine nestling	*Falco peregrinus*	3	0.02	99.63
European turtle dove	*Streptopelia turtur*	3	0.02	99.66
Yellowhammer	*Emberiza citrinella*	3	0.02	99.68
Budgerigar	*Melopsittacus undulates*	2	0.02	99.69
Common chiffchaff/willow warbler	*Phylloscopus collybita/P. trochilus*	2	0.02	99.71
Common linnet	*Linaria cannabina*	2	0.02	99.73
Meadow pipit	*Anthus pratensis*	2	0.02	99.74
Eurasian oystercatcher	*Haematopus ostralegus*	2	0.02	99.76
Red‐legged partridge	*Alectoris rufa*	2	0.02	99.77
Common reed warbler	*Acrocephalus scirpaceus*	2	0.02	99.79
European robin	*Erithacus rubecula*	2	0.02	99.80
Ruddy turnstone	*Arenaria interpres*	2	0.02	99.82
Water rail	*Rallus aquaticus*	2	0.02	99.84
Eurasian whimbrel	*Numenius phaeopus*	2	0.02	99.85
Pied avocet	*Recurvirostra avosetta*	1	0.01	99.86
Bar‐tailed godwit	*Limosa lapponica*	1	0.01	99.87
Eurasian bullfinch	*Pyrrhula pyrrhula*	1	0.01	99.87
Coal tit	*Periparus ater*	1	0.01	99.88
Dunnock	*Prunella modularis*	1	0.01	99.89
Field vole	*Microtus agrestis*	1	0.01	99.90
Green sandpiper	*Tringa ochropus*	1	0.01	99.91
Redwing	*Turdus iliacus*	1	0.01	99.91
Common reed bunting	*Emberiza schoeniclus*	1	0.01	99.92
Sandwich tern	*Thalasseus sandvicensis*	1	0.01	99.93
Eurasian siskin	*Spinus spinus*	1	0.01	99.94
Spotted flycatcher	*Muscicapa striata*	1	0.01	99.95
Eurasian teal	*Anas crecca*	1	0.01	99.95
Unidentified bat	Vespertilionidae or Rhinolophidae	1	0.01	99.96
Unidentified gull	*Larus* spp. or *Chroicocephalus* sp.	1	0.01	99.97
Unidentified pipit	*Anthus* spp.	1	0.01	99.98
Unidentified tern	*Sterna* spp.	1	0.01	99.98
Wood sandpiper	*Tringa glareola*	1	0.01	99.99
Western yellow wagtail	*Motacilla flava*	1	0.01	100.00

**TABLE A3:** Summary of the mass of prey (*n* = 9064) fed to peregrine nestlings across the study period 2019–2023; excludes cached and unidentified prey that could not be assigned to a size category.

Species/type		Biomass (kg)	% of total biomass	Cumulative %
Feral pigeon	*Columba livia*	656.70	44.88	44.88
Common starling	*Sturnus vulgaris*	264.08	18.05	62.92
Loft pigeon	*Columba livia*	166.20	11.36	74.28
Unidentified pigeon	*Columba* spp. or *Steptopelia* spp.	139.80	9.55	83.83
Unidentified (large)	N/a	55.80	3.81	87.65
Rose‐ringed parakeet	*Psittacula krameri*	33.14	2.26	89.91
Unidentified (medium)	N/a	24.75	1.69	91.60
Common wood pigeon	*Columba palumbus*	17.96	1.23	92.83
Unidentified corvid	*Corvus* spp. or *Coloeus* sp.	17.36	1.19	94.01
Eurasian collared dove	*Streptopelia decaocto*	13.12	0.90	94.91
Western jackdaw	*Coloeus monedula*	12.32	0.84	95.75
Common swift	*Apus apus*	7.70	0.53	96.28
Unidentified (small)	N/a	5.89	0.40	96.68
Common blackbird	*Turdus merula*	5.13	0.35	97.03
Common moorhen	*Gallinula chloropus*	3.96	0.27	97.30
Stock dove	*Columba oenas*	3.90	0.27	97.57
Rook	*Corvus frugilegus*	3.41	0.23	97.80
Common tern	*Sterna hirundo*	2.99	0.20	98.01
House sparrow	*Passer domesticus*	2.98	0.20	98.21
Black‐headed gull	*Chroicocephalus ridibundus*	2.40	0.16	98.37
Great spotted woodpecker	*Dendrocopos major*	2.30	0.16	98.53
Squab	*Columba* spp.	1.40	0.10	98.63
Unidentified wader	*Calidris* spp. or *Arenaria* sp.	1.35	0.09	98.72
Black‐tailed godwit	*Limosa limosa*	1.28	0.09	98.81
Red knot	*Calidris canutus*	1.22	0.08	98.89
Eurasian oystercatcher	*Haematopus ostralegus*	1.08	0.07	98.96
Eurasian jay	*Garrulus glandarius*	0.99	0.07	99.03
Eurasian whimbrel	*Numenius phaeopus*	0.96	0.07	99.10
Red‐legged partridge	*Alectoris rufa*	0.95	0.06	99.16
Eurasian magpie	*Pica pica*	0.91	0.06	99.22
Northern lapwing	*Vanellus vanellus*	0.69	0.05	99.27
Little grebe	*Tachybaptus ruficollis*	0.65	0.04	99.31
Common redshank	*Tringa totanus*	0.59	0.04	99.35
Common snipe	*Gallinago gallinago*	0.55	0.04	99.39
European greenfinch	*Chloris chloris*	0.54	0.04	99.43
Mistle thrush	*Turdus viscivorus*	0.50	0.03	99.46
Common cuckoo	*Cuculus canorus*	0.47	0.03	99.50
Common quail	*Coturnix coturnix*	0.42	0.03	99.52
European turtle dove	*Streptopelia turtur*	0.42	0.03	99.55
Eurasian chaffinch	*Fringilla coelebs*	0.35	0.02	99.58
European goldfinch	*Carduelis carduelis*	0.35	0.02	99.60
Sanderling	*Calidris alba*	0.34	0.02	99.62
Bar‐tailed godwit	*Limosa lapponica*	0.34	0.02	99.65
Dunlin	*Calidris alpina*	0.33	0.02	99.67
Song thrush	*Turdus philomelos*	0.33	0.02	99.69
Eurasian teal	*Anas crecca*	0.33	0.02	99.71
Common ringed plover	*Charadrius hiaticula*	0.32	0.02	99.74
Unidentified thrush	*Turdus* spp.	0.31	0.02	99.76
Unidentified gull	*Larus* spp. or *Chroicocephalus* sp.	0.30	0.02	99.78
Pied avocet	*Recurvirostra avosetta*	0.28	0.02	99.80
Water rail	*Rallus aquaticus*	0.26	0.02	99.81
Sandwich tern	*Thalasseus sandvicensis*	0.25	0.02	99.83
Ruddy turnstone	*Arenaria interpres*	0.24	0.02	99.85
White wagtail	*Motacilla alba*	0.23	0.02	99.86
Great tit	*Parus major*	0.18	0.01	99.88
Common kingfisher	*Alcedo atthis*	0.16	0.01	99.89
Eurasian skylark	*Alauda arvensis*	0.15	0.01	99.90
Peregrine egg	*Falco peregrinus*	0.14	0.01	99.91
Western house martin	*Delichon urbicum*	0.13	0.01	99.92
Unidentified tern	*Thalasseus* sp. or *Sterna* spp.	0.13	0.01	99.92
Unidentified passerine	Passeriformes	0.12	0.01	99.93
Peregrine chick	*Falco peregrinus*	0.12	0.01	99.94
Eurasian blackcap	*Sylvia atricapilla*	0.09	0.01	99.95
Yellowhammer	*Emberiza citrinella*	0.09	0.01	99.95
Green sandpiper	*Tringa ochropus*	0.09	0.01	99.96
Wood sandpiper	*Tringa glareola*	0.09	0.01	99.97
Redwing	*Turdus iliacus*	0.06	< 0.01	99.97
Budgerigar	*Melopsittacus undulates*	0.06	< 0.01	99.97
Common linnet	*Linaria cannabina*	0.04	< 0.01	99.98
Meadow pipit	*Anthus pratensis*	0.04	< 0.01	99.98
Field vole	*Microtus agrestis*	0.04	< 0.01	99.98
European robin	*Erithacus rubecula*	0.04	< 0.01	99.98
Eurasian blue tit	*Cyanistes caeruleus*	0.03	< 0.01	99.99
Unidentified bat	Vespertilionidae or Rhinolophidae	0.03	< 0.01	99.99
Common reed warbler	*Acrocephalus scirpaceus*	0.03	< 0.01	99.99
Eurasian bullfinch	*Pyrrhula pyrrhula*	0.02	< 0.01	99.99
Dunnock	*Prunella modularis*	0.02	< 0.01	99.99
Common reed bunting	*Emberiza schoeniclus*	0.02	< 0.01	99.99
Unidentified pipit	*Anthus* spp.	0.02	< 0.01	99.99
Common chiffchaff/willow warbler	*Phylloscopus collybita* or *P. trochilus*	0.02	< 0.01	100.00
Western yellow wagtail	*Motacilla flava*	0.02	< 0.01	100.00
Spotted flycatcher	*Muscicapa striata*	0.02	< 0.01	100.00
Eurasian siskin	*Spinus spinus*	0.01	< 0.01	100.00
Coal tit	*Periparus ater*	0.01	< 0.01	100.00

*Note:* The mass of each individual species or type of prey is represented by ‘Mass (g)’. ‘Total mass (kg)’ is the sum of the mass × the frequency of species or type of prey eaten (see Table A1 in Appendix 1).
